# Supplementary figures and images for: Twitter reveals human mobility dynamics during the COVID-19 pandemic
Source: PLoS One. 2020 Nov 10;15(11):e0241957. doi: 10.1371/journal.pone.0241957 (PMC7654838; doi:10.1371/journal.pone.0241957)

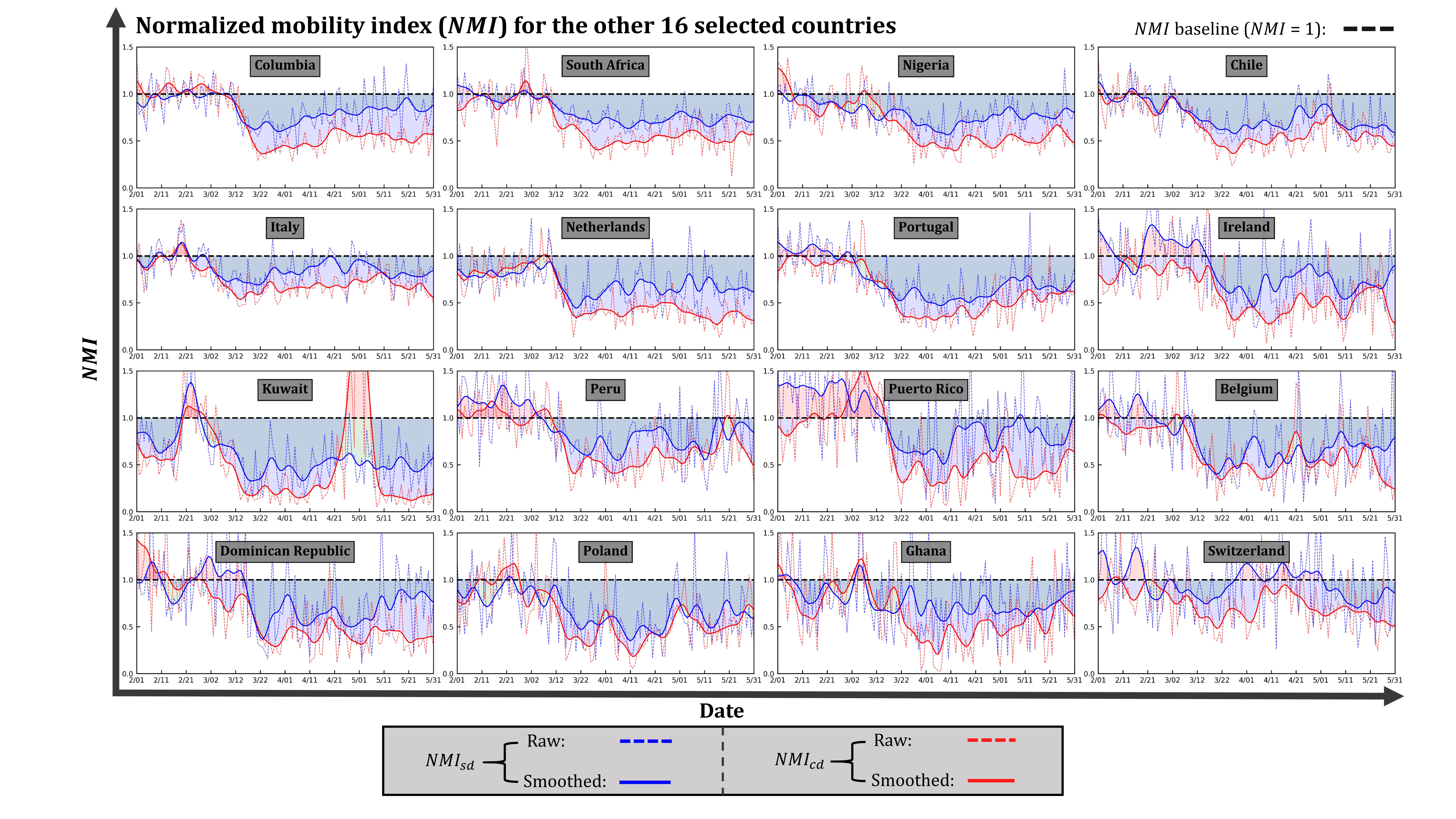

Supplement: S1 Fig — (TIF) [file pone.0241957.s001.tif]

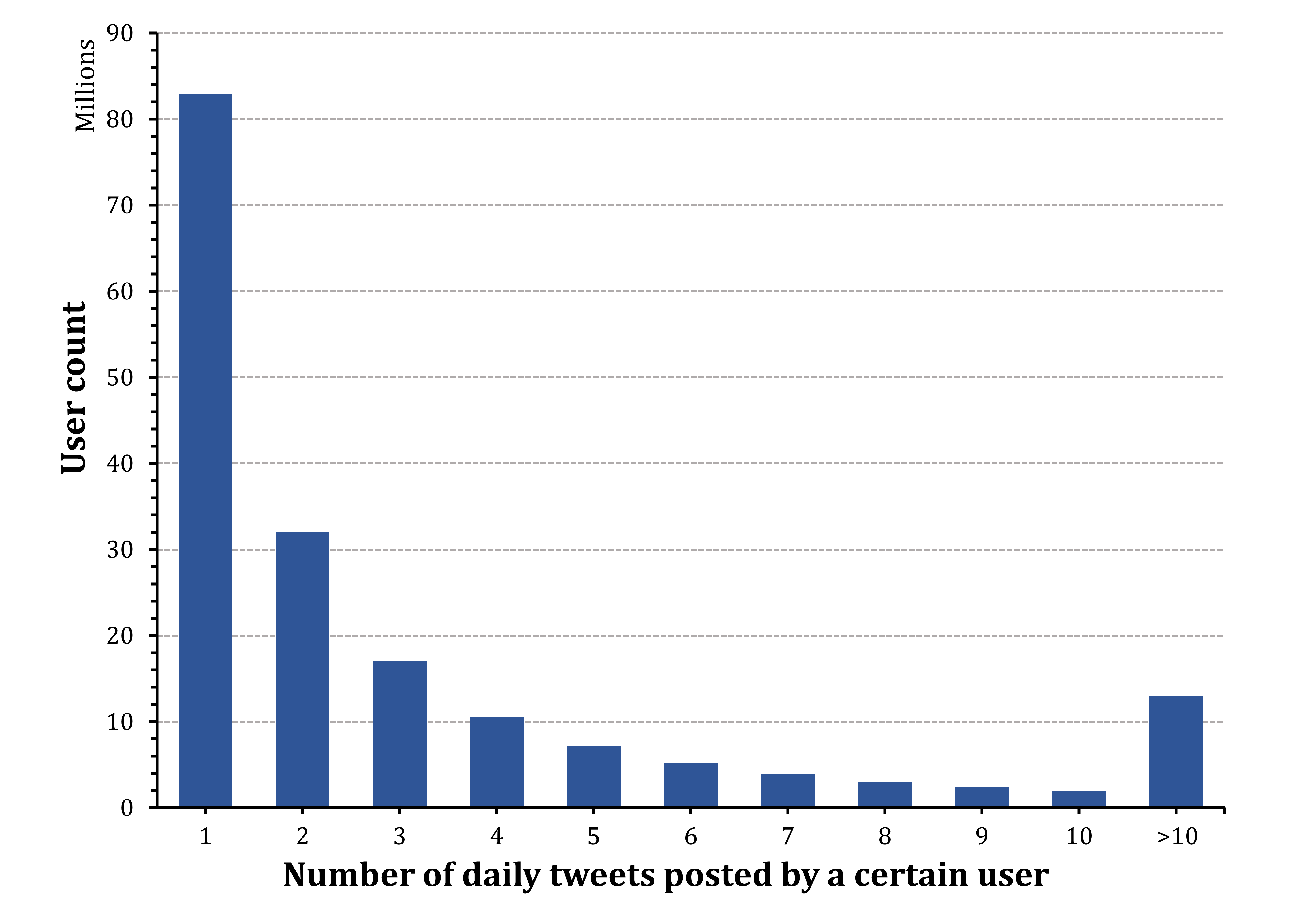

Supplement: S2 Fig — (TIF) [file pone.0241957.s002.tif]
