# Supplementary material for: Twitter reveals human mobility dynamics during the COVID-19 pandemic
Source: PLoS One. 2020 Nov 10;15(11):e0241957. doi: 10.1371/journal.pone.0241957 (PMC7654838; doi:10.1371/journal.pone.0241957)
Supplement: S1 Table — (DOCX) [file pone.0241957.s003.docx]

| Country names | User counts for single-day distance | User counts for cross-day distance |
| --- | --- | --- |
| The United States | 16,707,802 | 16,489,710 |
| Japan | 4,186,807 | 4,182,038 |
| Russia | 355,013 | 346,472 |
| United Kingdom | 3,689,886 | 3,678,842 |
| Saudi Arabia | 1,521,814 | 1,481,134 |
| Brazil | 11,434,280 | 11,357,493 |
| Turkey | 1,137,899 | 1,032,697 |
| India | 2,414,398 | 2,148,570 |
| Indonesia | 2,946,946 | 2,810,828 |
| Mexico | 1,806,742 | 1,846,731 |
| France | 1,048,109 | 1,056,299 |
| Spain | 1,838,289 | 1,905,574 |
| Canada | 1,045,614 | 1,084,076 |
| Thailand | 696,204 | 646,429 |
| Philippines | 2,988,538 | 2,827,301 |
| Germany | 482,896 | 494,020 |
| South Korea | 163,637 | 155,588 |
| Argentina | 1,739,256 | 1,740,652 |
| Australia | 514,958 | 515,739 |
| Malaysia | 1,172,728 | 1,129,547 |
| Columbia | 1,042,175 | 1,059,255 |
| South Africa | 819,243 | 753,783 |
| Nigeria | 1,022,576 | 869,998 |
| Chile | 543,235 | 538,647 |
| Italy | 552,595 | 578,308 |
| Netherlands | 346,032 | 353,696 |
| Portugal | 309,264 | 309,246 |
| Ireland | 293,315 | 302,301 |
| Kuwait | 298,786 | 273,592 |
| Peru | 228,072 | 227,383 |
| Puerto Rico | 151,911 | 153,208 |
| Belgium | 151,182 | 155,700 |
| Dominican Republic | 142,339 | 142,090 |
| Poland | 135,801 | 137,329 |
| Ghana | 207,690 | 187,394 |
| Switzerland | 79,178 | 82,563 |

**S1 Table. Country names and accumulated user counts for distance calculation in selected countries.**
