# Supplementary material for: Twitter reveals human mobility dynamics during the COVID-19 pandemic
Source: PLoS One. 2020 Nov 10;15(11):e0241957. doi: 10.1371/journal.pone.0241957 (PMC7654838; doi:10.1371/journal.pone.0241957)
Supplement: S2 Table. State abbreviations, state names, and accumulated user count for distance calculation in the CONUS states — (DOCX) [file pone.0241957.s004.docx]

**S2 Table. State abbreviations, state names, and user counts for distance calculation in CONUS states.**

| State abbreviations | State name | User count for single-day distance | User count for cross-day distance |
| --- | --- | --- | --- |
| AL | Alabama | 214,122 | 207,922 |
| AR | Arkansas | 91,668 | 90,675 |
| AZ | Arizona | 431,698 | 444,767 |
| CA | California | 3,269,827 | 3,330,049 |
| CO | Colorado | 263,890 | 269,059 |
| CT | Connecticut | 159,283 | 165,477 |
| DC | District of Columbia | 195,241 | 214,213 |
| DE | Delaware | 29,701 | 29,495 |
| FL | Florida | 1,231,773 | 1,183,890 |
| GA | Georgia | 632,721 | 581,886 |
| IA | Iowa | 121,218 | 122,483 |
| ID | Idaho | 46,676 | 47,508 |
| IL | Illinois | 713,377 | 734,515 |
| IN | Indiana | 319,111 | 323,587 |
| KS | Kansas | 123,075 | 132,023 |
| KY | Kentucky | 165,500 | 167,184 |
| LA | Louisiana | 406,025 | 397,695 |
| MA | Massachusetts | 377,953 | 388,301 |
| MD | Maryland | 413,685 | 425,821 |
| ME | Maine | 28,455 | 27,340 |
| MI | Michigan | 401,920 | 399,389 |
| MN | Minnesota | 213,127 | 211,893 |
| MO | Missouri | 245,810 | 250,894 |
| MS | Mississippi | 114,020 | 109,112 |
| MT | Montana | 19,333 | 19,317 |
| NC | North Carolina | 525,344 | 522,276 |
| ND | North Dakota | 20,790 | 21,164 |
| NE | Nebraska | 86,780 | 89,106 |
| NH | New Hampshire | 42,273 | 43,094 |
| NJ | New Jersey | 502,807 | 543,953 |
| NM | New Mexico | 83,299 | 93,251 |
| NV | Nevada | 309,223 | 316,065 |
| NY | New York | 1,388,111 | 1,404,096 |
| OH | Ohio | 651,672 | 656,250 |
| OK | Oklahoma | 181,377 | 183,555 |
| OR | Oregon | 209,813 | 217,102 |
| PA | Pennsylvania | 576,512 | 574,684 |
| RI | Rhode Island | 59,523 | 61,941 |
| SC | South Carolina | 181,945 | 174,734 |
| SD | South Dakota | 22,600 | 22,985 |
| TN | Tennessee | 393,817 | 399,912 |
| TX | Texas | 2,517,699 | 2,506,707 |
| UT | Utah | 126,219 | 130,322 |
| VA | Virginia | 429,465 | 431,457 |
| VT | Vermont | 15,367 | 15,767 |
| WA | Washington | 378,476 | 381,998 |
| WI | Wisconsin | 164,438 | 164,377 |
| WV | West Virginia | 54,373 | 55,928 |
| WY | Wyoming | 11,919 | 12,879 |
